# Prevalence and correlates of transactional sex among women of low socioeconomic status in Portland, OR

**DOI:** 10.1186/s12905-020-01088-1

**Published:** 2020-10-02

**Authors:** Timothy W. Menza, Lauren Lipira, Amisha Bhattarai, Victoria Cali-De Leon, E. Roberto Orellana

**Affiliations:** 1 HIV/STD/TB Section, Public Health Division, Oregon Health Authority, 800 NE Oregon Street, Portland, Oregon 97232 USA; 2 Oregon Health & Sciences University (OHSU), Portland, Oregon USA; 3 Multnomah County Health Department, Portland, Oregon USA; 4 Portland State University, Portland, Oregon USA

**Keywords:** Transactional sex, Adverse childhood experiences, HIV testing, Pre-exposure prophylaxis, Substance use

## Abstract

**Background:**

Women who report transactional sex are at increased risk for HIV and other sexually transmitted infections (STIs). However, in the United States, social, behavioral, and trauma-related vulnerabilities associated with transactional sex are understudied and data on access to biomedical HIV prevention among women who report transactional sex are limited.

**Methods:**

In 2016, we conducted a population-based, cross-sectional survey of women of low socioeconomic status recruited via respondent-driven sampling in Portland, Oregon. We calculated the prevalence and, assessed the correlates of, transactional sex using generalized linear models accounting for sampling design. We also compared health outcomes, HIV screening, and knowledge and uptake of HIV pre-exposure prophylaxis (PrEP) between women who did and did not report transactional sex.

**Results:**

Of 334 women, 13.6% reported transactional sex (95% confidence interval [CI]: 6.8, 20.5%). Women who reported transactional sex were older, more likely to identify as black, to identify as lesbian or bisexual, to experience childhood trauma and recent sexual violence, and to have been homeless. Six percent (95% CI: 1.8, 10.5%) of women with no adverse childhood experiences (ACEs) reported transactional sex compared to 23.8% (95% CI: 13.0, 34.6%) of women who reported eleven ACEs (*P* <  0.001). Transactional sex was strongly associated with combination methamphetamine and opiate use as well as condomless sex. Women who reported transactional sex were more likely to report being diagnosed with a bacterial STI and hepatitis C; however, HIV screening and pre-exposure prophylaxis knowledge and use were low.

**Conclusions:**

In a sample of women of low socioeconomic status in Portland, Oregon, transactional sex was characterized by marginalized identities, homelessness, childhood trauma, sexual violence, substance use, and sexual vulnerability to HIV/STI. Multi-level interventions that address these social, behavioral, and trauma-related factors and increase access to biomedical HIV prevention are critical to the sexual health of women who engage in transactional sex.

## Background

The incidence of HIV in the United States (U.S.) has remained stable in the past few years and HIV diagnoses among women have been in decline [1]. However, certain subpopulations of women (e.g., women of color, women with low income or low education, and women who inject drugs) are still at considerable risk for HIV [2]. Research on social and structural forces that increase vulnerability for HIV and other sexually transmitted infections (STIs) among women has demonstrated that a multitude of factors, including minority status, low socioeconomic status (SES), and adverse experiences, intersect to create milieus of risk where women, especially women of color, are at a disadvantage when it comes to avoiding HIV and STI [2, 3].

Women who engage in transactional sex may be at particularly high risk for HIV infection. Transactional sex, or sex exchanged for money, goods, services, and/or status, is a complex phenomenon [4]. A 2016 meta-analysis of 14 studies of women who reported exchanging sex for money or drugs in the U.S. found a pooled HIV prevalence of 17.3% [5], compared to a prevalence of 0.16% in U.S. women [1].

Estimates of the prevalence of transactional sex among women in the U.S. range from 4 to 41% [6–12]. However, no studies have estimated the prevalence of transactional sex in the Pacific Northwest, a region with rising homelessness and injection drug use, factors strongly associated with transactional sex and recent increases in HIV infection [13–15].

Indeed, prior studies indicate that women with certain characteristics are more likely to engage in transactional sex that others. Compared to women who did not report transactional sex, those who did were more likely to use substances and to report recent criminal justice involvement [11]; to experience homelessness [7, 10]; and, to face sexual intimate and commercial partner violence [8, 9, 16]. Moreover, women who engage in transactional sex may also be more likely to have had early adverse experiences. Growing research indicates that trauma throughout the lifespan is associated with HIV-related risk factors, including drug use, mental health problems, homelessness, and condomless sex [17] and women living with HIV report higher rates of trauma and violence than the general population of U.S. women [18]. This overlap of transactional sex, trauma, and HIV-related vulnerability is likely most acute among women of low SES [8, 19, 20].

While many studies have demonstrated an association between transactional sex and HIV prevalence among women in the U.S., most of those studies were conducted over 20 years ago [5]. Since that time, there have been key advances in HIV prevention and treatment, including HIV treatment as prevention, undetectable equals un-transmittable, and HIV pre-exposure prophylaxis. Analysis of more current data is needed to better understand HIV risk and prevention behaviors and related health outcomes among women who engage in transactional sex in the modern era.

In the current study, we determined the prevalence and correlates of transactional sex among women of low SES in the Portland, Oregon metropolitan area. We also examined the relationship of transactional sex and HIV/STI-related prevention and health outcomes.

## Methods

We conducted a cross-sectional analysis of a sample of women of low SES as part of the 2016 Centers for Disease Control and Prevention (CDC) National HIV Behavioral Surveillance (NHBS) heterosexual cycle in the Portland, Oregon, metropolitan area [21, 22]. We focused our work on women of low SES due to well-documented intersections between poverty, transactional sex, and HIV-related risk behaviors [2, 3, 8, 19, 20, 23].

We recruited participants via respondent-driven sampling (RDS) [24]. Recruitment began with fifteen initial participants, or seeds. Eligible seeds who completed the survey were provided three to five coded coupons to recruit others (i.e., recruits). Eligible recruits completed the survey and, in turn, recruited three to five additional participants.

Per CDC protocol, participants were eligible if they were aged 18–60 years; identified as cis-gender female; resided in a census tract of the Portland-Hillsboro, Oregon-Vancouver, Washington, U.S. metropolitan statistical area (MSA); did not previously participate in the current survey cycle; were able to complete the survey in English or Spanish; reported sex with at least one opposite sex partner in the prior 12 months; and, reported an income below the federal poverty level or completed less than a high school education (i.e., low SES). Also, per CDC protocol, individuals who identified as transgender or had sex with a same sex partner, but not an opposite sex partner, in the prior 12 months were not eligible for participation.

Eligible participants completed an anonymous face-to-face computer-assisted survey that captured information about social, economic, and behavioral vulnerability to HIV infection and access to HIV testing, care, and prevention. Participants were remunerated for their participation ($50 for completing the interview and $25 for rapid HIV testing).

### Measures

#### Transactional sex

We created a binary variable that categorized participants who reported receiving money or drugs for sex from one or more casual sex partners in the prior 12 months as having engaged in transactional sex. Participants were not asked if they received money or drugs for sex from main sex partners.

#### HIV testing, HIV prevention, and health outcomes

We assessed HIV testing, knowledge of HIV PrEP, and use of PrEP in the prior 12 months. We also asked participants whether they had been ever diagnosed with hepatitis C and whether they had been diagnosed with gonorrhea, chlamydia, or syphilis in the prior 12 months.

#### Socio-demographics

The survey instrument captured age, race/ethnicity, sexual orientation, education, employment, income, homelessness, and incarceration history.

#### Trauma

We used the 11-item Adverse Childhood Experiences (ACEs) questionnaire to assess experiences of emotional, physical, and sexual abuse (2 items); physical and emotional neglect; parental separation or divorce; and, household substance use, mental illness, partner violence, and incarceration prior to age 18 [25]. To assess the cumulative effects of ACE, we tallied the number of ACE endorsed by each participant to create a continuous variable ranging from zero to eleven. We also inquired about sexual intimate partner violence in the prior 12 months.

#### Substance use

We inquired about injection drug use and non-injection use of methamphetamine and opiates (i.e., heroin, prescription opioid pain medications) in the past 12 months.

#### Sexual behavior

We asked women to enumerate their sexual partners and queried whether they had had condomless vaginal or anal sex with a casual partner in the prior 12 months.

### Statistical analyses

#### Prevalence of transactional sex

As each participant had a different sampling probability based on their network size, we calculated Gile successive sampling (SS) weights for each participant using RDS Analyst [26, 27]. We based our weights on the American Community Survey (ACS) 2011–2015 population estimates of people aged 18–64 living below the federal poverty level in the Portland-Hillsboro, Oregon-Vancouver, Washington, U.S. MSA (161,186 individuals) [28]. We estimated that 82% were sexually active in the prior 12 months [29]. Thus, our base population for weight calculations was 0.82*161,186 = 132,173. We calculated weighted medians and interquartile ranges (IQRs) and proportions and bootstrap 95% confidence intervals (CIs) for continuous and categorical variables, respectively.

### Correlates of transactional sex

To determine correlates of transactional sex, we first compared characteristics of women who engaged in transactional sex to characteristics of women who did not engage in transactional sex. We used design-based chi-squared tests and tests of medians to compare categorical and continuous variables, respectively. Then, we ran multivariable analyses. To accommodate a potentially small number of women reporting transactional sex and avoid an overfit multivariable model [30], we limited our number of potential covariates to the ten that we thought would be most highly associated with transactional sex: age, race/ethnicity, sexual orientation, homelessness, incarceration, ACEs score, sexual violence, injection drug use, methamphetamine and opiate use, and condomless vaginal or anal sex with a casual partner in the prior 12 months.

We created four multivariable models. Model 1 included age, race/ethnicity, sexual orientation, homelessness, and incarceration. Model 2 added ACEs score and sexual violence to Model 1. Model 3 added substance use associated with transactional sex to Model 2. Model 4 added sexual behavior associated with transactional sex to Model 3. We used generalized linear models with a log link and a Poisson distribution to estimate risk ratios (RRs) and bootstrap 95% CIs. Multivariable models were adjusted for network size, used sampling weights, and calculated standard errors based on clustered sampling by recruitment chain.

We computed the variance inflation factor (VIF) and tolerance (1/VIF) for each of the ten predictors included in Model 4 to assess for collinearity [31]. All tolerance values were less than 0.1, indicating that each predictor was unlikely to be a linear combination of the others.

### Engagement in HIV testing and prevention, and health outcomes

We compared the binary outcomes of HIV testing, hearing about and taking PrEP, and diagnoses of hepatitis C and bacterial STI between women who reported transactional sex and those who did not using design-based chi-squared tests.

We used RDS Analyst [26] and STATA 15.1 (College Station, TX) for all analyses with statistical significance defined as *P* <  0.05.

## Results

As part of the 2016 NHBS heterosexual cycle in Portland, we screened 385 women of whom 334 (87%) were eligible and completed the survey. Of the 51 who were not eligible to participate, 31 (61%) did not report a male sex partner, 9 (18%) were previous participants, 7 (14%) were over age 60, and 3 (6%) were not alert enough to complete the survey (categories not mutually exclusive).

The median age of eligible women was 35 years (IQR: 23, 46; Table 1). Sixty-eight percent were women of color, 42.9% percent were black. Most identified as heterosexual, while 1.4% identified as lesbian and 16.4% as bisexual. Almost 40% had been homeless in the prior 12 months. Only one-third were employed and almost one-fourth did not complete high school. Forty-three percent reported ever being incarcerated. Eighty-one percent were living in poverty.

**Table 1:** Selected characteristics of women who did and did not report transactional sex in the prior 12 months among women participating in National HIV Behavioral Surveillance, Portland, Oregon, United States, 2016 ^a^

	Total, %	No transactional sex, %	Transactional sex, %	*P* value
Socio-demographics				
Age, median (IQR)	35 (23, 46)	34 (23–45)	40 (30–46)	0.066
Race/ethnicity				0.395
White	32.0 (22.6, 41.0)	29.5 (20.2, 38.9)	45.9 (20.7, 71.2)	
Black	42.9 (34.5, 51.2)	44.3 (35.0, 53.6)	34.2 (13.1, 55.2)	
Latina/x	8.3 (4.2, 12.5)	7.9 (4.0, 11.8)	11.2 (1.9, 28.7)	
Other, multiracial	17.0 (10.8, 23.2)	18.3 (12.1, 24.5)	8.7 (3.1, 17.7)	
Sexual orientation				< 0.001
Heterosexual	82.2 (75.6, 88.7)	88.4 (84.0, 92.7)	42.9 (20.0, 65.8)	
Lesbian, bisexual	17.8 (10.2, 26.8)	11.6 (7.3, 16.0)	57.1 (34.2, 80.0)	
Homeless, past 12 months				< 0.001
No	63.3 (55.3, 71.3)	69.8 (61.6, 77.9)	22.6 (5.6, 39.7)	
Yes	36.7 (28.2, 45.1)	30.2 (22.1, 38.4)	77.4 (60.3, 94.4)	
Ever incarcerated				< 0.001
No	57.4 (48.0, 65.1)	64.1 (55.5, 71.9)	17.8 (7.8, 35.7)	
Yes	42.6 (34.1, 51.1)	35.9 (28.1, 44.4)	82.2 (64.3, 92.2)	
Trauma				
ACE score, median (IQR)	4 (2–7)	4 (2–7)	7 (6–9)	< 0.001
Sexual intimate partner violence, past 12 months				< 0.001
No	85.8 (79.2, 91.6)	92.3 (86.5, 95.7)	45.9 (24.2, 69.1)	
Yes	14.2 (8.9, 21.7)	7.7 (4.3, 13.5)	54.2 (30.9, 75.8)	
Substance use and sexual behaviors				
Ever used drugs by injection				< 0.001
No	84.5 (78.8, 90.7)	91.9 (85.5, 94.5)	43.1 (22.8, 66.1)	
Yes	14.5 (8.2, 20.8)	8.1 (4.4, 14.5)	56.9 (33.8, 77.2)	
Methamphetamine and opiate use, past 12 months				< 0.001
None	74.2 (67.1, 81.3)	82.1 (75.6, 87.2)	25.8 (10.8, 44.1)	
Methamphetamine only	10.8 (4.5, 17.2)	8.0 (4.9, 12.9)	29.9 (7.6, 58.9)	
Opiates only	6.8 (3.8, 9.9)	5.8 (2.9, 11.5)	14.5 (3.6, 25.3)	
Methamphetamine and opiates	7.3 (3.0, 11.7)	3.9 (2.0, 7.5)	29.8 (6.3, 53.3)	
Condomless vaginal or anal sex with a casual partner, past 12 months				< 0.001
No	59.4 (51.9, 67.0)	68.0 (59.7, 76.2)	5.4 (1.3, 12.1)	
Yes	40.6 (32.8, 48.3)	32.0 (23.7, 40.3)	94.6 (87.9, 99.9)	

*ACE* Adverse childhood experience, *CI* Confidence interval, *IQR* Interquartile range

^a^All data are percentages (95% CI) unless otherwise noted; medians and percentages are weighted

Ninety-two percent of women reported ≥1 ACEs, 70.7% reported ≥3 ACEs, and 10.5% reported ≥9 ACEs; median ACE score was 4 [2–7]. Fourteen percent experienced sexual intimate partner violence in the prior 12 months. Fourteen percent had ever used drugs by injection and 26% used non-injection methamphetamine, opiates, or both. Women reported a median of 2 sexual partners (IQR: 1–3) and 40.6% reported condomless vaginal or anal sex with a casual partner.

Of the 334 women, 241 (65.1%) reported testing HIV-negative prior to survey participation, 2 (0.3%) reported testing HIV-positive, and 94 (34.6%) had never been tested or did not know their results. Based on point-of-care HIV testing, 329 (99.6%) results were negative, one (0.3%) was positive, and four (0.1%) were indeterminate or missing.

### Prevalence and correlates of transactional sex

Approximately 14% of women reported transactional sex in the prior 12 months (13.6, 95% CI: 6.8, 20.5%). Women who reported transactional sex were less likely to identify as heterosexual, and more likely to report homelessness and incarceration (Table 1). Those who reported transactional sex reported also more ACEs and were more likely to experience recent sexual violence. Women who reported transactional sex were more likely to use drugs by injection and use non-injection methamphetamine, opiates, or both. Women who reported transactional sex were more likely to report condomless sex with a casual partner.

In Model 1, women who were older, black, bisexual or lesbian, experienced homelessness in the prior 12 months, and were ever incarcerated were more likely to report transactional sex (Table 2). In Models 2 and 3, women who were older, black, lesbian or bisexual, experienced homelessness in the prior 12 months, and who had a greater number of ACEs and who experienced sexual violence were more likely to report transactional sex.

**Table 2:** Multivariable models of correlates of transactional sex among women participating in National HIV Behavioral Surveillance, Portland, Oregon, United States, 2016

	Model 1			Model 2			Model 3			Model 4		
	aRR	95% CI	*P* value	aRR	95% CI	*P* value	aRR	95% CI	*P* value	aRR	95% CI	*P* value
Age	**1.03**	**1.01, 1.07**	**0.014**	**1.04**	**1.01, 1.06**	**0.003**	**1.04**	**1.01, 1.07**	**0.019**	**1.03**	**1.01, 1.06**	**0.039**
Race/ethnicity												
White	REF			REF			REF			REF		
Black	**1.87**	**1.01, 3.48**	**0.048**	**3.16**	**1.70, 5.87**	**< 0.001**	**3.96**	**2.11, 7.46**	**< 0.001**	**3.60**	**2.17, 6.00**	**< 0.001**
Latina/x	1.58	0.59, 4.20	0.360	1.17	0.52, 2.64	0.703	1.33	0.62, 2.84	0.460	1.13	0.54, 2.38	0.742
Other, multiracial	0.72	0.27, 1.94	0.515	0.97	0.34, 2.76	0.949	1.03	0.40, 2.63	0.954	0.98	0.39, 2.46	0.969
Lesbian, bisexual (v heterosexual)	**4.21**	**2.36, 7.82**	**< 0.001**	**2.71**	**1.44, 5.10**	**0.002**	**2.14**	**1.19, 3.85**	**0.011**	**1.68**	**1.06, 2.69**	**0.028**
Homeless, past 12 months	**3.53**	**1.86, 6.68**	**< 0.001**	**2.74**	**1.62, 4.63**	**< 0.001**	**2.53**	**1.50, 4.25**	**< 0.001**	**2.65**	**1.62, 4.34**	**< 0.001**
Ever incarcerated	**2.80**	**1.08, 7.31**	**0.035**	1.98	0.78, 5.01	0.152	1.63	0.62, 4.27	0.319	1.70	0.71, 4.08	0.233
ACE score				**1.25**	**1.10, 1.41**	**< 0.001**	**1.20**	**1.07, 1.34**	**0.001**	**1.12**	**1.04, 1.22**	**0.005**
Sexual intimate partner violence, past 12 months				**3.33**	**1.76, 6.31**	**< 0.001**	**2.84**	**1.62, 5.00**	**< 0.001**	**2.07**	**1.24, 3.46**	**0.006**
Ever used drugs by injection							1.69	0.86, 3.30	0.126	1.44	0.77, 2,68	0.250
Methamphetamine and opiate use, past 12 months												
None							REF			REF		
Methamphetamine only							1.52	0.66, 3.44	0.328	1.26	0.61, 2.62	0.534
Opiates only							1.76	0.70, 4.42	0.229	1.54	0.67, 3.55	0.310
Methamphetamine and opiates							**2.82**	**1.38, 5.76**	**0.004**	**2.45**	**1.44, 4.14**	**0.001**
Condomless anal or vaginal sex with a casual partner, past 12 months										**8.71**	**2.31, 32.7**	**0.001**

*ACE* Adverse childhood experience, *aRR* Adjusted risk ratio, *CI* Confidence interval

In Model 4, a one-year increase in age was associated with a 3% (95% CI: 1–7%) increase in the probability of reporting transactional sex comparing older women to younger women. Black women were more likely to report transactional sex compared to their white counterparts. For a one-point increase in the ACEs score, the probability of reporting transactional sex was 12% (95%: 3–21%) higher comparing women with a higher score to women with a lower score. Women who reported recent sexual violence were two times more likely to report transactional sex compared to women who did not report sexual violence. Women who used both methamphetamine and opiates were more likely to report transactional sex compared to women who used neither. Reporting condomless sex with a casual partner was associated with a 9-fold higher probability of transactional sex.

### Adverse childhood experiences and transactional sex

**Fig. 1:**
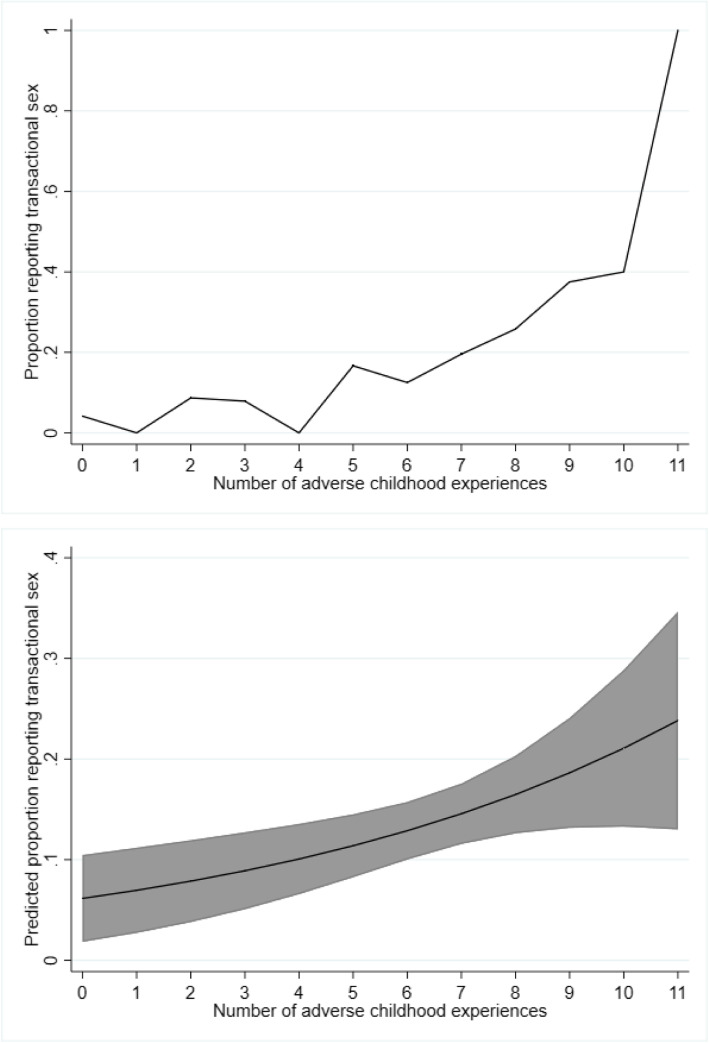
Actual (**a**) and predicted (**b**) proportion of women reporting transactional sex according to number of adverse childhood experiences, National HIV Behavioral Surveillance, Portland, Oregon, United States, 2016

In a post hoc analysis, we examined the crude and predicted proportions of women reporting transactional sex associated with each ACE score (Fig. 1). The predicted proportion of women reporting transactional sex was 6.1% (95% CI: 1.8, 10.5%) for an ACEs score of zero compared to 23.8% (95% CI: 13.0, 34.6%) for an ACEs score of 11.

Women who reported transactional sex were more likely to report each of the 11 ACEs queried. However, compared to women who did not report transactional sex women who reported transactional sex were statistically significantly more likely to report that they lived with someone who was depressed, mentally ill or suicidal (37.6% v. 69.9%, *P* = 0.005); that a parent or adult in their home swore at them, insulted them, or put them down (58.4% v. 83.3%, *P* = 0.017); and, that someone at least 5 years older than the respondent or an adult touched them sexually (42.9% v. 82.2%, *P* <  0.001), tried to make the respondent touch them sexually (29.2% v. 71.1%, *P* <  0.001), or forced the respondent to have sex (26.3% v. 77.9%, *P* <  0.001).

### HIV prevention behaviors and health outcomes

Less than a third of women in the total sample reported an HIV test in the prior 12 months (Table 3) and there was no difference in testing comparing women who did and did not report transactional sex. Only 3.9% had heard of PrEP and 0.8% had taken PrEP in the prior 12 months. There was no difference in PrEP knowledge between women who did and did not report transactional sex, but statistically significantly fewer women who reported transactional sex reported taking PrEP in the prior 12 months compared to women who did not report transactional sex (0% v. 1.0%, *P* <  0.001). In contrast, 29.1% of those who reported transactional sex reported a hepatitis C diagnosis compared to 6.1% of those who did not report transactional sex (*P* = 0.001). Women who reported transactional sex were twice as likely to report a diagnosis of a bacterial STI in the past 12 months compared to women who reported no transactional sex, but this difference was not statistically significant (*P* = 0.132). Only one woman in the sample tested HIV-positive by rapid testing and she did not report transactional sex.

**Table 3:** HIV testing, knowledge and use or pre-exposure prophylaxis, and diagnosis of hepatitis C and bacterial sexually transmitted infection among women participating in National Behavioral Surveillance, Portland, Oregon, United States, 2016 ^a^

Transactional Sex	HIV test, past 12 months, % (95% CI)	Heard of PrEP, ever, % (95% CI)	Took PrEP, past 12 months, % (95% CI)	Diagnosed HCV, ever, % (95% CI)	Bacterial STI, past 12 months, % (95% CI)
No	25.5 (19.1, 31.9)	3.5 (1.2, 5.8)	1.0 (0.2, 4.0)	5.9 (2.3, 9.5)	4.5 (1.7, 7.4)
Yes	22.6 (5.1, 40.1)	5.6 (1.5, 12.7)	0.0	28.8 (7.1, 50.6)	10.7 (0.1, 21.2)
Total	25.1 (18.8, 31.4)	3.8 (1.6, 6.0)	0.8 (0.2, 1.9)	9.0 (4.5, 13.6)	5.4 (2.7, 8.0)
*P* value	0.761	0.532	< 0.001	0.001	0.132

^a^ All data are weighted percentages. *CI* Confidence interval, *HCV* Hepatitis C virus, *PrEP* Pre-exposure prophylaxis, *STI* Sexually transmitted infection

Healthcare access does not seem to explain testing and PrEP access outcomes. Of the entire sample, 90.4, 91.0, and 88.6% of the sample had health insurance, had a regular source of care, and saw a healthcare provider in the prior year, respectively. There were no statistically significant differences in these variables between women who did and did not report transactional sex.

## Discussion

In a sample of low-SES women from the Portland, Oregon metropolitan area, 13.6% reported receiving money or drugs for sex in the prior 12 months. This estimate of transactional sex is similar to studies with similar inclusion/exclusion criteria conducted on the West Coast of the U.S. [7–9] but lower than estimates in U.S. East Coast samples [10–12] and a national U.S. sample [6]. Similar to prior studies, Black women, older women, and women who reported sexual violence were more likely to report transactional sex [7, 9, 10, 12, 16].

Childhood trauma was pervasive among the women in our sample. Over 90% of women experienced ≥1 ACE, an estimate significantly higher than recent prevalence estimates among U.S. adults [32]. Furthermore, ACEs were associated with an increased probability of transactional sex independent of the effects of demographics, incarceration, homelessness, substance use, sexual behavior, and recent sexual intimate partner violence. Experiences of childhood sexual abuse were not the only ACEs associated with transactional sex; women who reported transactional sex were more likely to experience emotional abuse and to report living with a family member with mental illness. We observed a dose-response relationship between ACEs and the likelihood of transactional sex.

Our findings are consistent with the 2012 Behavioral Risk Factor Surveillance Survey (BRFSS), wherein women who experienced more ACEs were more likely to report a composite outcome of HIV risk behavior that included exchange sex [17] and a study of South African women which found an association between a different childhood trauma score and transactional sex [33, 34]. Similar dose-response relationships have been found with myriad other behaviors and physical and mental health outcomes [35]. The development of life course-based, public health interventions to address the effects of trauma and harness the resilience that can come from surviving trauma are essential [36].

Women who reported both opioid and methamphetamine use were more likely to report transactional sex than women who used neither or either substance. In Oregon and other U.S. jurisdictions, there has been increasing overlap of methamphetamine and opioid use since at least 2011 [37–39]. Use of opioids and methamphetamine may produce a desirable, synergistic high; women who have experienced trauma may only be able to have sex while on methamphetamine; methamphetamine may mitigate opioid withdrawal symptoms; methamphetamine may be less stigmatized and easier to obtain; methamphetamine may allow people to function to complete daily task; and/or, methamphetamine may be used as a form of currency [40]. The implications of this overlap for sexual health require further investigation.

Despite clear indications for frequent HIV testing and PrEP, access to, and uptake of, biomedical prevention among women who reported transactional sex was limited. Self-reported condomless sex with a casual partner, hepatitis C infection, and bacterial STI were nine, five, and two times more likely among women who reported transactional sex, respectively. However, among women who reported transactional sex, only 23% tested for HIV in the past year, 6% had ever heard of PrEP, and none had taken PrEP. Research indicates that women vulnerable to HIV infection view PrEP as an important HIV prevention option, but may not be hearing about PrEP from their providers [41]. These data behoove medical and public health communities to develop programs to increase knowledge of, and access to, screening and biomedical HIV prevention as part of comprehensive sexual health services for HIV-vulnerable women.

Our study has several limitations. First, NHBS is cross-sectional; we cannot infer a causal relationship between the examined vulnerabilities, behaviors, and health outcomes and transactional sex. Second, the assessment of ACEs and other variables was retrospective and subject to recall bias [42]. Third, the definition of transactional sex is too narrow and does not capture the range of practices that comprise transactional sex. Thus, the study may underestimate the prevalence of transactional sex and the context of the transactional sex practices captured in this study may not generalize to the context of other transactional sex practices. Fourth, the sample of women reporting transactional sex was relatively small, limiting our statistical models to a select set of variables to avoid overfitting. Fifth, while RDS strives to yield a probability sample of the target population, existing estimators rely heavily on the assumptions of the underlying network structure and of how that network is sampled [43]. Therefore, we cannot be certain that the sample we recruited truly represents the underlying population of women of low SES in Portland, Oregon. Furthermore, we recruited a racially diverse population rarely represented in Portland, Oregon, but, again, our sample may not be representative of the area.

## Conclusion

In a sample of women of low SES in Portland, Oregon, transactional sex was characterized by marginalized identities, homelessness, childhood trauma, sexual violence, substance use, and sexual vulnerability to HIV/STI. Public health efforts to reduce HIV-related health disparities and health inequities among women in the U.S. are unlikely to be effective if the social and structural factors that increase vulnerability to HIV/STIs are not addressed. Multi-level, combination prevention strategies that integrate empirically-based interventions with trauma informed care have the potential to not only reduce vulnerability of HIV/STI, but also alter the nature of the social and structural determinants of women’s health.

## Data Availability

The datasets for current study are available from the corresponding author on reasonable request.

## Abbreviations

ACE: Adverse childhood experience
ACS: American community survey
BRFSS: Behavioral risk factor surveillance survey
CI: Confidence interval
HIV: Human immunodeficiency virus
IQR: Interquartile range
MSA: Metropolitan statistical area
NHBS: National HIV behavioral surveillance
PrEP: Pre-exposure prophylaxis
RDS: Respondent driven sampling
RR: Risk ratio
SES: Socio-economic status
SS: Successive sampling
STI: Sexually transmitted infection
US: United States
