# Correction to: Prevalence and correlates of transactional sex among women of low socioeconomic status in Portland, OR

**DOI:** 10.1186/s12905-020-01098-z

**Published:** 2020-10-20

**Authors:** Timothy W. Menza, Lauren Lipira, Amisha Bhattarai, Victoria Cali-De Leon, E. Roberto Orellana

**Affiliations:** 1 HIV/STD/TB Section, Public Health Division, Oregon Health Authority, 800 NE Oregon Street, Portland, OR 97232 USA; 2 Oregon Health and Sciences University (OHSU), Portland, OR USA; 3 Multnomah County Health Department, Portland, OR USA; 4 Portland State University, Portland, OR USA

## Correction to: BMC Women’s Health (2020) 20:219 https://doi.org/10.1186/s12905-020-01088-1

Following publication of the original article [1], we were notified of a typo in the 4th paragraph of the “Statistical analyses” section:

- “We computed the variance inflation factor (VIF) and tolerance (1/VIF) for each of the ten predictors included in Model 4 to assess for collinearity. All tolerance values were **less** than 0.1, indicating that each predictor was unlikely to be a linear combination of the others.”

The word **less** should actually read **greater**.
